# Detection and Recognition of Fearful Facial Expressions During the Coronavirus Disease (COVID-19) Pandemic in an Italian Sample: An Online Experiment

**DOI:** 10.3389/fpsyg.2020.02252

**Published:** 2020-09-11

**Authors:** Federica Scarpina

**Affiliations:** ^1^“Rita Levi Montalcini” Department of Neurosciences, University of Turin, Turin, Italy; ^2^Istituto Auxologico Italiano, IRCCS, U.O. di Neurologia e Neuroriabilitazione, Ospedale S. Giuseppe, Piancavallo, Italy

**Keywords:** COVID-19, facial emotion recognition, fear, social cognition, implicit behavior, online assessment

## Abstract

In this observational study, the psychological ability to recognize the others’ fearful expressions in Italian individuals during the pandemic COVID-19 lockdown was explored through a behavioral task performed online. An implicit version of the traditional facial emotion recognition task, grounded on the attentional and unconscious mechanism of the *redundant target effect*, was used. The experiment was scripted through the free software OpenSesame (Mathôt et al., 2012) and published on the Internet through the free software Jatos (Lange et al., 2015). The Reaction Time and level of Accuracy in detecting fearful expressions were computed. Overall, the data of 86 Italian individuals were collected. When their performance was scored in terms of Reaction Time, the redundant target effect did not emerge; instead, the expected effect was observed when the level of Accuracy was considered. Overall, the performance registered in this Italian sample in terms of accuracy was in line with previous results reported in Scarpina et al. (2018), in which a long extended version of the same behavioral task was used in a traditional experimental setting. This study might offer some considerations regarding the adoption of online experiments – together with self-report surveys – to assess the psychological and behavioral functioning during social restriction measures.

## Introduction

The severe acute respiratory syndrome coronavirus 2 (COVID-19) generated a rapid and tragic health emergency worldwide. In this pandemic, Italy was hit very hard (Gatto et al., 2020; Remuzzi and Remuzzi, 2020), with 213.013 documented cases, with 29.315 deaths as of May 05, 2020.^1^ With the “*I stay at home*” (Io resto a casa) decree of 2020, March 9, the Italian government declared the entire national territory as a protected area (i.e., the lockdown): until May 04, 2020, people were requested to move only if necessary; also, the prohibition of assembly and closure of commercial activities was declared.

During the lockdown, people experienced social isolation and psychological burden as well as expressed negative emotions, such as fear, together with anger, and sadness. Overall, individuals reported anxiety- and depressive-related symptoms (Brooks et al., 2020; Li et al., 2020; Pfefferbaum and North, 2020; Xiao, 2020; Wang et al., 2020). Moreover, the restraining measures modified substantially lifestyles, social perception, and confidence in the institutions. Nevertheless, individual responses to the psychological distress might vary according to the individual psychological characteristics, such as affective temperament and attachment features (Moccia et al., 2020), but also according to the subjective understanding of the information from institutions and scientific panels, as well as from media and social media, on the pandemic and its consequences (Cinelli et al., 2020). Even though confinement and social isolation may strictly limit the interpersonal (physical) contact, during the COVID-19 pandemic, people were easily exposed to images and narrations with a higher emotional impact, as well as information about others’ behaviors and emotional reactions through the media and the social media. Moreover, information on the epidemic and the lives of other people, especially those affected with COVID-19, was easily obtained. Multiple technologies for the delivery of voice communications and multimedia sessions over internet protocol networks allowed individuals to communicate not only *verbally* but also *non-verbally* with others (relatives, colleagues, and friends). Nevertheless, during the quarantine, most of the individuals shared the physical space with their relatives and families, possibly for a longer time in comparison with the preceding living conditions. Thus, in the case of the COVID-19 pandemic, physical distancing did not necessarily mean emotional distancing.

How might researchers explore individuals’ psychological functioning during a lockdown, when face-to-face assessments were not allowed? Online surveys were generally used, as described in the recent works by Moccia et al. (2020) in the Italian context. This approach may offer the advantages of faster data collection, larger samples, and reductions in costs when compared with the most traditional sample collection methodologies (post, or phone); questionnaires allow collecting the subjective and explicit description of own psychological behavior (Scarpina et al., 2018). However, as in my knowledge, no previous study has proposed an online behavioral task to explore the psychological functioning in the case of social distancing. Therefore, in the present study I described the application of an *online* version of an implicit facial emotion recognition task focused on the emotion of fear (Scarpina et al., 2018) on an Italian sample during the COVID-19 epidemic lockdown. This task allowed registering the individuals’ behavior when they were exposed to fearful expressions.

**FIGURE 1:**
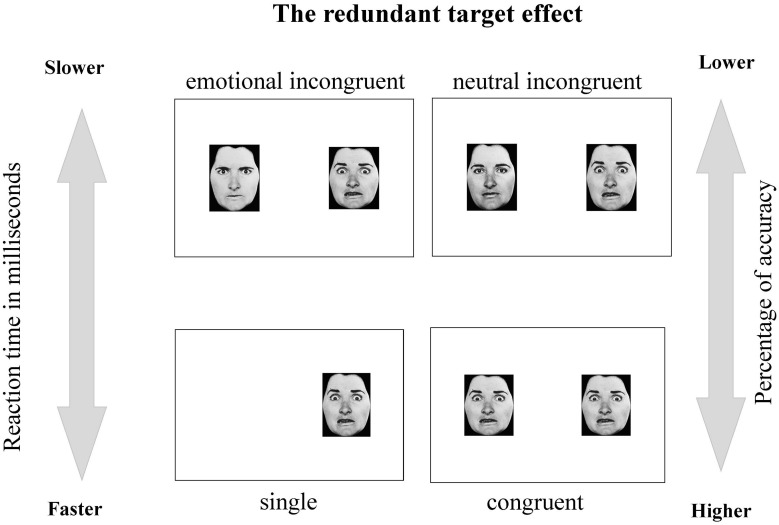
Schematic representation of the attentional mechanism of the redundant target effect. For each experimental condition, an example of the visual stimulus was shown. According to the effect, participants would be faster **(left arrow)** and more accurate **(right arrow)** to provide their answer in the single and congruent conditions, in comparison with the emotional incongruent and neutral incongruent conditions, in which they were generally slower and less accurate.

The facial emotion recognition task has a long-tradition in psychology: *emotional sensitivity* (Domes et al., 2009) as well as *emotional contagion* (de Gelder et al., 2004; Moody et al., 2007; Werner and Gross, 2010) can be assessed through the measurement of individuals’ ability to decode and label the emotion expressed by others. Human faces are a powerful channel of non-verbal communication, mediating social interaction, empathy, and psychological functioning: through facial expressions, all human emotions can be communicated to the others and automatically, rapidly, and implicitly decoded (Vuilleumier et al., 2002). Thus, once an emotion is recognized, people may efficiently adjust their behavior (Ekman, 1992). In 2018, Scarpina et al. (2018) described an implicit version of the traditional facial emotion recognition, which assesses the participants’ behavior according to the very well-known attentional mechanism of the “*redundant target effect*” (Miniussi et al., 1998; Diano et al., 2017) applied to the facial expressions (Tamietto et al., 2006, 2007; Tamietto and de Gelder, 2008, 2010; Won and Jiang, 2013). Since this cognitive attentional phenomenon occurs at a very early level of the visual processing, it is not related to a decisional or premotor mechanism (Miniussi et al., 1998; Iacoboni and Zaidel, 2003); so, in other words, it is an implicit and automatic process. The attentional effect exploits in a specific behavior relative to the stimuli detection (i.e., the Reaction Time), as shown on the left side of Figure 1: people respond faster when two identical targets (i.e., two faces expressing the emotion of fear) are presented simultaneously rather than when presented alone (i.e., one fearful face). Moreover, the competitive presence of a non-identical stimulus (a face expressing another emotion, such as anger, or a neutral expression) affects the velocity in detecting the target. Even though the redundant target effect was traditionally described for the stimulus detection (Miniussi et al., 1998), it was also reported at the level of accuracy in recognizing correctly the target (Tamietto et al., 2006, 2007; Scarpina et al., 2018; Figure 1, right side), representing the ability to discriminate different emotional expressions. Thus, higher levels of accuracy are generally registered in the case of two identical targets or the target alone (i.e., one fearful face) in comparison with the condition in which it is shown together with a competitive non-identical stimulus (Scarpina et al., 2018).

In this research, I focused on the emotion of fear. As primary emotion, it is very critical for human survival. Fear is generally described as a motivational state aroused by specific threatening stimuli that give rise to defensive behavior or escape (McFarland, 1981). When we recognize the emotion of fear in the others’ facial expression, it works as an alert of a possible external danger with which we have to deal. Phenomenologically, fear is linked to anxiety (Steimer, 2002), which represents a generalized response to an unknown threat or intrapersonal psychic conflict (Craig et al., 1995). Because in the case of an epidemic the external danger (i.e., the virus) is not visible, the others’ expression and behavior may be an important clue about the presence of a possible threat. Nevertheless, it was established that the observation of others’ verbal and non-verbal behaviors may be crucial in experiencing fear. Indeed, fears can be acquired and learned through *direct* experience or *indirectly* through social transmission. Interestingly, these two processes share neural mechanisms, in which there amygdala is the core (Olsson and Phelps, 2007; Debiec and Olsson, 2017), even in the case of fearful stimuli that are not consciously perceived or attentionally detected (Öhman et al., 2007).

The implicit facial emotion recognition task (Scarpina et al., 2018) allows quantifying the participants’ behavior; in other words, it might allow providing an experimental answer to the following question: how do they react when they are confronting with fearful expressions? The redundant target effect in the case of fearful facial expressions was consistently reported in healthy individuals; moreover, it was also observed as altered in those clinical conditions characterized by a dysfunctional emotional processing (see Diano et al., 2017 review). Thus, in this study, the aim was to explore if the individuals’ behavior at the implicit facial emotion recognition task delivered online would mirror the previous evidence relative to the redundant target effect in healthy individuals (Miniussi et al., 1998; Tamietto et al., 2006, 2007; Tamietto and de Gelder, 2008, 2010; Won and Jiang, 2013; Scarpina et al., 2018).

## Materials and Methods

This study was approved by the Bioethics Commission of the University of Turin (Italy). It was performed accordingly to the Declaration of Helsinki’s principles (World Medical Association, 1991). The entire study was scripted through the free software OpenSesame (Mathôt et al., 2012). It was published on the Internet through the free software Jatos (Lange et al., 2015) and run on a web server hosted in an AWS public cloud. In my knowledge, at the time of this experiment, the webOS Open Source Edition 2.0^2^ and its interaction with the free software Jatos was experimental and still under development. The experiment was ran only on laptop and personal computer (thus, no smarthphone or tablet). The experiment was long, which was around 5 min. Participants’ recruitment was performed via social media pages. The link for the experiment was available from April 12nd, 2020 to May 3rd, 2020 (the day before the start of the Italian “*phase 2*,” when in Italy social restrictions changed).

### Participants

All participants were volunteers who provided informed consent electronically as part of the web experiment. They were free to withdraw at any time closing the browser, and were naïve to the rationale of the study. Participants were not remunerated for their participation. Only Italian participants were enrolled in this study. For each participant, demographic and social information – as described in Table 1 – was collected.

**TABLE 1:** Sample’ demographical characteristics.

Percentage	Statistical results
**Gender**	
24% males	χ^2^ = 4.63; *p* < **0.001**
75% females	
**Handedness**	
83.7% right-handed	χ^2^ = 99.37**; *p* < 0.001**
12.8% left-handed	
3.5% ambidextrous	
**Age (range)**	
19–30 years: 41.9%	χ^2^ = 35.48; ***p* < 0.001**
31–45 years: 39.5%	
46–60 years: 14%	
61 years and over: 4.7%	
**Level of education**	
8 years: 3.5%	χ^2^ = 34.34; ***p* < 0.001**
13 years: 24.4%	
16 years: 9.3%	
18 years: 39.5%	
More than 18 years: 6%	
**Living condition during the lockdown**	
Alone: 10.5%	χ^2^ = 32.95; ***p* < 0.001**
Spouse/partner: 36%	
Spouse/partner and children: 20.9%	
Original family: 30.2%	
Roommates: 2.3%	
**COVID-19-related symptoms**	
No symptoms declared: 69.8%	χ^2^ = 3.55; ***p* < 0.001**
Not sure: 30. 2%	
Certain diagnosis: 0%	
**Involvement in care activities**	
Involved: 23.3%	χ^2^ = 66.72; ***p* < 0.001**
Not involved: 73.3%	
Not sure: 3.5%	

*For each demographical component, the percentage of respondents for each class was reported. The statistical result relative to the chi-square (χ^2^) test used to determine a statistically significant difference between the observed frequencies in the categories/classes of each demographical component was reported. In bold, when the p-value was significant (≤ 0.05). N = 86.*

Also, respondents answered a short survey according to a four-point Likert scale questionnaire exploring the subjective perception of their own psychological functioning and the level of empathy, at the time of the experiment. Details were reported in Table 2.

**TABLE 2:** Questions on the psychological functioning.

	% of respondents				Statistical results
	1 – not at all	2 – not much	3 – somewhat	4 – very much	
*In this moment*					
*I feel calm*	2.3%	23.3%	72.1%	2.3%	χ^2^ = 111.76; ***p* < 0.001**
*I feel tense*	20.9%	61.6%	17.4%	0%	χ^2^ = 31.14; ***p* < 0.001**
*I feel upset*	17.4%	65.1%	17.4%	0%	χ^2^ = 39.09; ***p* < 0.001**
*I feel relaxed*	17.4%	24.4%	54.7%	3.5%	χ^2^ = 48.14; ***p* < 0.001**
*I feel happy*	11.6%	39.5%	44.2%	4.7%	χ^2^ = 40.32; ***p* < 0.001**
*I feel worried*	2.3%	55.8%	39.5%	2.3%	χ^2^ = 75.3; ***p* < 0.001**
*I feel emphatic*	1.2%	19.8%	53.5%	25.6%	χ^2^ = 48.41; ***p* < 0.001**
*I feel feelings that I cannot identify*	51.2%	32.6%	16.3%	0%	χ^2^ = 15.72 ***p* < 0.001**
*People around me appear more anxious/afraid than usually.*	11.6%	51.2%	26.7%	10.5%	χ^2^ = 37.07; ***p* < 0.001**

*Answers were provided on the four-step (from 1 to 4) Likert scale. For each of the four-step Likert scale, the percentage (%) of respondents was reported. The statistical result relative to the chi-square (χ^2^) test used to determine a statistically significant difference between the observed frequencies in the steps for each psychological question was reported. In bold, when the p-value was significant (≤0.05). N = 86.*

### Experimental Task

A short version of the implicit facial emotion recognition task (Scarpina et al., 2018) focused on the emotion of fear was used. It was a go–no go task. Photographs of male and female faces with a fearful expression were shown in four different experimental conditions: (*i*) *single*: the fearful face was presented on the right OR left of a fixation cross; (*ii*) *congruent*: the fearful face was presented simultaneously on the right AND left of the fixation cross; (*iii*) *emotional incongruent*: the fearful face was presented on the right OR left of the fixation cross along with a different negative emotion (i.e., anger), or (*iv*) *neutral incongruent*: the target was presented on the right OR left of the fixation cross along with a neutral expression (Figure 1). For each experimental condition, eight trials were shown, with 32 valid trials overall. Moreover, eight catch trials (two for each experimental condition) were randomly presented. Overall, the task consisted of 40 trials. In each trial, pictures were shown for 350 ms; participants had a maximum of 1500 ms from the onset of the visual stimuli to provide an answer. The inter-stimulus interval varied randomly between 650 and 950 ms. Participants were required to respond as soon as they noticed a fearful expression, pressing a key (i.e., the letter *h*) on the PC keyboard.

## Analyses

### Demographic Information and the Psychological Questions

The χ^2^-test was used to test any differences in the observed frequencies.

### Experimental Task

Individuals who reported more than four *false alarms* (i.e., they answered in the case of a catch trial, meaning when no target was shown) were excluded from the sample. Also, answers provided over the threshold of 1000 ms and below the threshold of 50 ms were not considered in the analyses. The *Reaction Time* (RT) in ms from the stimulus onset relative to the valid trials (i.e., when the target, meaning the emotion of fear, was correctly detected) and the level of Accuracy (i.e., the percentage of correct answers to the valid trials) were computed for each of the four experimental conditions. Independently for RT and percentage of accuracy, a repeated-measure ANOVA with the within-factors of *Condition* (single, congruent, emotional incongruent, neutral incongruent) was run to probe the main hypothesis of this study. Estimated marginal mean comparisons Bonferroni-corrected were applied in the case of a significant main effect. Successively, in the case of a significant main effect of *Condition*, the same analysis was performed introducing each demographical component (expressed as nominal variables) to verify the possible significant interaction with the within-subject factor of *Condition*. Finally, in the case of a significant main effect of *Condition* in the previous main analyses, the repeated-measure ANOVA with the within-factors of *Condition* (single, congruent, emotional incongruent, neutral incongruent) would be computed again, introducing the score at each psychological question (independently investigated) as a covariate, to assess the effect of the psychological state on the main behavior.

### Comparison With Previous Data

For both the RT and the level of Accuracy, an independent sample *t*-test was performed independently for each experimental condition between the performance registered in this experiment and the performance reported in Scarpina et al. (2018), in which 25 healthy subjects (16 women, age *M* = 42 years; *SD* = 14; range 23–61; education *M* = 15; *SD* = 2; range: 8–18) were tested with a long extended version of the task. Specifically, this previous version consisted of overall 384 trials (32 valid trials and 16 catch trials for each experimental condition; each condition was tested twice). The timing of picture presentation and the inter-stimulus interval were the same as that of the short version presented in this study. Moreover, for each comparison, a Bayes factor was calculated (Rouder et al., 2009) to express preference for either the null hypothesis (no difference between the two samples’ behavior) or the alternative hypothesis (the two groups reported a different behavior).

## Results

### Participants

Overall, the data of 86 Italian individuals were collected. Thus, the sample size was larger in comparison with previous studies on the redundant target effect, such as *n* = 25 in Scarpina et al. (2018), *n* = 25 in Tamietto et al. (2006, experiment 2); *n* = 25 in Tamietto and de Gelder (2008). In Table 1, the sample’s demographical characteristics were extensively reported. In Table 2, the percentage of answers relative to the psychological questions was reported. Also, the results on the statistical analyses relative to the demographical characteristics (Table 1) and the psychological questionnaire’s ratings (Table 2) were reported.

### Experimental Task

#### RT

**FIGURE 2:**
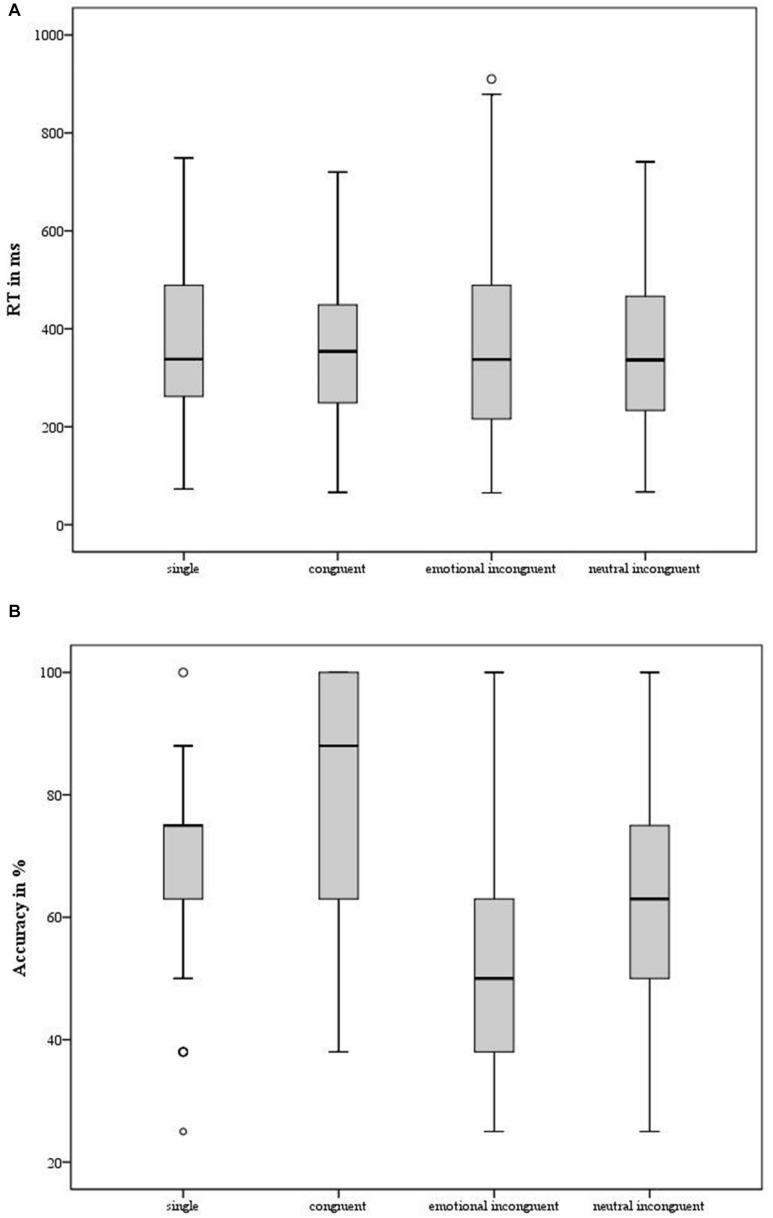
Implicit facial emotion recognition task. **(A)** For each experimental condition (*x*-axis: single, congruent, emotional incongruent, neutral incongruent), the mean of Reaction Time expressed in millisecond (*y*-axis – ms) was reported. The minimum, the lower quartile, the median, the upper quartile, the maximum, and the outliers were shown. According to the main analyses, no difference emerged between the experimental conditions. **(B)** For each experimental condition (*x*-axis: single, congruent, emotional incongruent, neutral incongruent), the mean of the level of Accuracy expressed in percentage (*y*-axis – %) was shown. Again, the minimum, the lower quartile, the median, the upper quartile, the maximum, and the outliers were shown. According to the main analyses, significant differences emerged between conditions, mirroring the redundant target effect.

No significant main effect of *Condition* emerged [*F*(3, 243) = 0.26; *p* = 0.85; partial η^2^ = 0.003]: as shown in the Figure 2A, participants detected fearful expression at the same speed, independently from the experimental conditions. In different words, no redundant target effect in the RTs emerged.

Because there was no main effect for *Condition*, no further analysis on the RT was performed.

#### Accuracy

A significant main effect for *Condition* emerged [*F*(3, 204) = 36.18; *p* < 0.001; partial η^2^ = 0.34]. The *post hoc* comparisons showed a significant different level of Accuracy between all the experimental conditions (*p* ≤ 0.008), except for the comparison of single condition vs. neutral incongruent condition (*p* = 0.056). Specifically, as shown in Figure 2B, individuals reported a significantly higher level of Accuracy in the congruent condition and in the single condition in comparison with the emotional incongruent condition and the neutral incongruent condition, in line with the redundant target effect, as described in Figure 1.

Successively, the interaction with the sample’s demographical characteristics was investigated. A significant interaction emerged only in the case of the between-subject factor of *Education* [*F*(12, 192) = 2.06; *p* = 0.02; partial η^2^ = 0.11], suggesting a different level of Accuracy within the experimental conditions in relation to the different levels of education. Specifically, when the *post hoc* comparisons were performed, no significant difference emerged between the different levels of education for the *single* condition (*p* ≥ 0.16). For the *congruent* condition, a significant difference emerged between individuals reporting 13 years of attended schooling (*M* = 100; *SD* = 0) and those with more than 18 years (*M* = 92.31; *SD* = 10.01; *p* = 0.018), with no other significant difference (*p* ≥ 0.23). When the *emotional incongruent* condition was analyzed, no significant difference emerged between the different levels of education (*p* ≥ 0.55). Finally, for the *neutral incongruent* condition, a significant difference emerged between the individuals that reported 18 years of attended schooling (*M* = 56.22; *SD* = 10.84) and individuals with more than 18 years (*M* = 76.18; *SD* = 13.35; *p* = 0.11), with no other significant difference (*p* = 1). No other significant interaction (*p* ≥ 0.06) emerged.

Successively, the effect of the psychological state on the level of Accuracy was investigated. Only when the score relative to the question “*People around me appear more anxious/afraid than usually*” was introduced as covariate in the analyses did a significant interaction with *Condition* emerge [*F*(3, 201) = 2.72; *p* = 0.04; partial η^2^ = 0.003], in the absence of a significant main effect of the covariate [*F*(1, 67) = 0.045; *p* = 0.83; partial η^2^ = 0.001] (single corrected *M* = 70.17; *SD* = 1.84; congruent corrected *M* = 81.14; *SD* = 2.55; emotional incongruent corrected *M* = 56.42; *SD* = 2.41; neutral incongruent corrected *M* = 64.59; *SD* = 2.38). For the other psychological questions, no main effect of covariate or a significant interaction emerged (*p* > 0.05).

#### Comparisons With Previous Data

**FIGURE 3:**
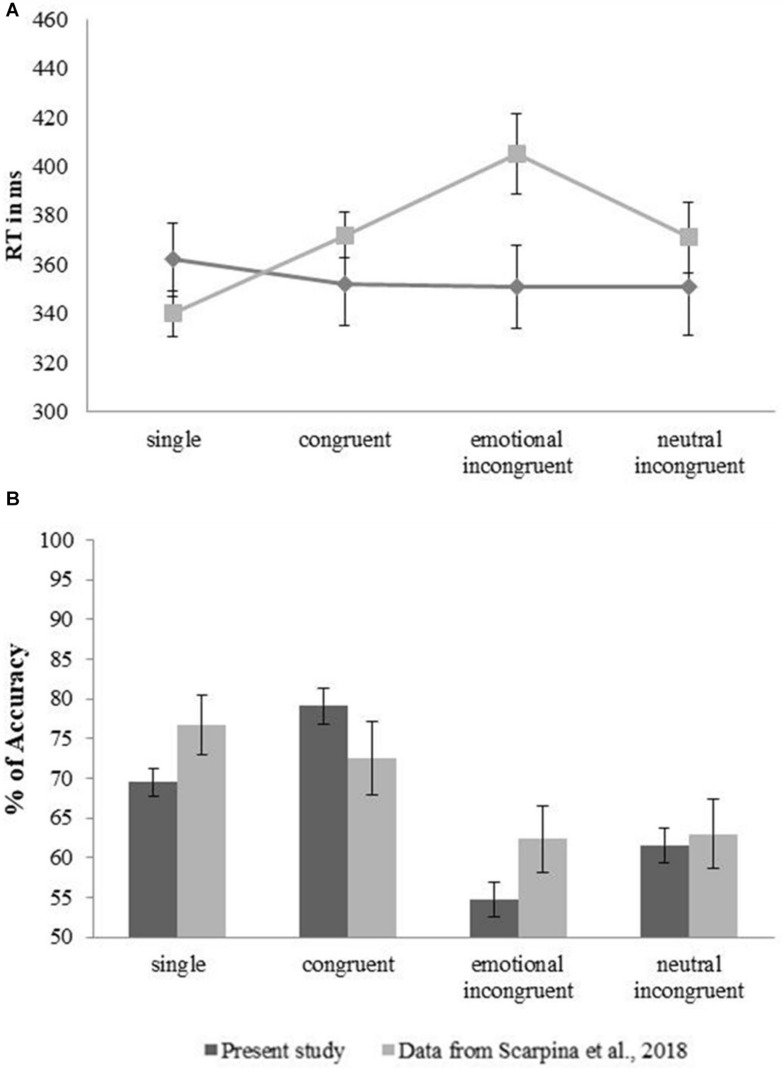
Comparison with the data reported in Scarpina et al. (2018). **(A)** The mean (lines) and standard error (vertical lines) relative to Reaction Time expressed in milliseconds (*y*-axis – ms) for each experimental condition (*x*-axis: single, congruent, emotional incongruent, neutral incongruent) was reported for the sample (*n* = 86) of the present experiment (dark gray lines) and the sample (*n* = 20) in Scarpina et al. (2018) (light gray lines). **(B)** The mean (bars) and the standard error (vertical lines) relative to the level of Accuracy expressed in percentage (*y*-axis – %) for each experimental condition (*x*-axis: single, congruent, emotional incongruent, neutral incongruent) were reported for the sample (*n* = 86) of the present experiment (dark gray bars) and the sample (*n* = 20) in Scarpina et al. (2018) (light gray bars).

In Figure 3, the *RT* (left part) and the level of *Accuracy* (right part) registered in this experiment in each experimental condition were shown in comparison with the data reported in Scarpina et al. (2018). In Table 3, the statistical results relative to the comparisons between these two samples were reported.

**TABLE 3:** Statistical comparison with the data reported in Scarpina et al. (2018).

		Reaction time in milliseconds				Level of accuracy in percentage			
		Single	Congruent	Emotional incongruent	Neutral incongruent	Single	Congruent	Emotional incongruent	Neutral incongruent
**Present study *n* = 86**	M	362	352	351	351	69.46	79.06	54.77	61.57
	*SD*	*139*	*156*	*157*	*185*	*15.6*	*21.58*	*19.99*	*20.23*
**Scarpina et al. (2018)*n* = 25**	M	340	372	405	371	76.62	72.5	62.31	63
	*SD*	*42*	*42*	*73*	*64*	*16.8*	*20.68*	*18.93*	*19.83*
df = 104	t	0.69	0.56	1.46	0.47	1.82	1.23	1.53	0.28
	*p*-value	0.48	0.57	0.13	0.63	0.07	0.22	0.12	0.77
	Cohen’s *d*	0.21	0.17	0.44	0.14	0.36	0.31	0.38	0.07
	95% CI	−40.49 to 84.49	−29.99 to 49.99	−125.54 to17.54	−103.43 to 63.43	−14.95 to 0.63	−3.98 to 17.1	−17.28 to 2.2	−11.35 to 8.49
	Bayes factor in favor of the hypothesis	1.56 null	1.5 null	1.25 null	1.43 null	1.28 **alternative**	1.35 null	1.1 null	1.22 null

*For each experimental condition (single, congruent, emotional incongruent, neutral incongruent), the mean (M) and standard deviation (SD) relative to Reaction Time (expressed in milliseconds–left part) and the level of Accuracy (expressed in percentage–right part) were shown for the sample of this study and the sample reported in Scarpina et al. (2018). To verify any possible difference between the data relative to the two samples, an independent sample t-test was used; the results (i.e., t-value, p-value, degrees of freedom (df), Cohen’s d, and the 95% standard symmetric confidence interval (CI)) were reported. In bold, any significant result (p-value ≤ 0.05). Since no significant difference emerged, the Bayes factor was calculated and here reported. Also, the preference in confirming the null hypothesis (i.e., no difference between the samples) or the alternative hypothesis (i.e., a difference between the samples, in bold) was reported.*

Overall, the results relative to the independent sample *t*-tests suggested no difference between the two samples’ behavior. When the Bayes factor was computed, a preference in confirming the null hypothesis was formulated almost for all comparisons, except for the comparison relative to the percentage of accuracy reported in the single condition.

## Discussion

This research aimed to explore the psychological ability in detecting and recognizing fearful expressions in an Italian sample, during the lockdown in the COVID-19 pandemic, through an online experiment. To this aim, the implicit facial emotion recognition task (Scarpina et al., 2018) grounded on the attentional mechanism of the *redundant target effect* (Miniussi et al., 1998; Tamietto et al., 2006, 2007; Tamietto and de Gelder, 2008, 2010; Won and Jiang, 2013) was delivered on the Internet through the free software Jatos (Lange et al., 2015).

When the performance was described in terms of Reaction Time, representing the index relative to the ability in detecting fearful stimuli, the expected *redundant target effect* was not observed. Individuals reported a similar reaction time in all four experimental conditions, independently from the concurrent presence of another emotional or neutral stimulus, as shown in Figure 2A. This result noticeably contrasted with large previous evidence (such as Miniussi et al., 1998; Iacoboni and Zaidel, 2003; Tamietto et al., 2007; Tamietto and de Gelder, 2008; Scarpina et al., 2018) (for a review on the topic, Diano et al., 2017), according to which the speed of processing (i.e., the reaction time) is reported to be significantly different between the experimental conditions. Specifically, the reaction time in the case of simultaneous but incongruent (emotional and neutral) emotional stimuli was generally reported to be slower in comparison with the case of congruent or single stimuli, as shown in Figure 1. However, when the data collected in the present experiment was compared with the data reported in Scarpina et al. (2018), collected through an extended version of the task run in a traditional experimental setting before the pandemic, no difference in the behavioral performances emerged. However, this absence of a difference between them might be due to the larger standard deviation of the data distribution observed in the data collected through the online version in comparison with Scarpina et al. (2018) (Table 3). Indeed, some cautions should be necessary for interpreting this result. Indeed, when an experimental task is run online, technical criticisms (that cannot be solved remotely) in terms of timing (such as the accurate timing of visual stimuli presentation, or of the subjective responses) and mostly related to the participants’ bandwidth should be considered. The discussion of such timing issues is out of the scope of the present manuscript; however, further comments on the constraints of online behavioral tasks were reported by Crump et al. (2013) and, more recently, by Anwyl-Irvine et al. (2020). Because of the criticisms on the RT, it is highly recommendable to rate the individual’s performance accordingly to an index (such as the percentage of the level of Accuracy) registered independently from the timing. Crucially, the redundant target effect was observed when the sample’s performance was assessed in terms of the level of Accuracy: individuals were more accurate in recognizing fear when expressed by two identical faces, or only one face, in comparison to the condition in which the emotion was presented together with a face expressing another emotion, such as anger, or a neutral expression. When the sample’ performance of this study was compared with the results reported in Scarpina et al. (2018), no difference emerged. The results relative to the level of Accuracy seemed to suggest a preserved ability in recognizing fearful expression; instead, the results relative to Reaction Time appeared to be less clear. Interestingly, the level of Accuracy in recognizing correctly others’ fearful expressions seemed to be related to the respondents’ subjective perception of the others’ emotional functioning (i.e., *how the others appeared to me*), rather than by the self-description relative to their own psychological functioning (i.e., *how I feel*). In this experiment, few questions were used to investigate explicitly the subjective psychological functioning; instead, no clinical psychological questionnaires were adopted, because of two technical issues. First, the use of extended psychological questionnaires would cause an increase in the time frame of the experiment. Moreover, while the software OpenSesame (Mathôt et al., 2012) allows implementing questionnaires, some technical criticisms emerged in the interaction with the software Jatos (Lange et al., 2015). Nevertheless, these technical criticisms might be weighted considering that the software adopted in this study allowed me to propose an open-source tool. Another criticism of the present study might be the shortness of the task presented in this paper in comparison with the long version (384 trials) of the original task (Scarpina et al., 2018). Even though a higher number of trials might be preferable to test an attentional mechanism (likewise *the redundant target effect*), a task longer than 40 trials would dramatically increase the risk of dropout or decrease the subjective level of vigilance and concentration over time (Crump et al., 2013; Anwyl-Irvine et al., 2020). When a traditional test is tuned in a computerized version, it should be considered at a new different test (Bauer et al., 2012); however, no further test to verify the replicability and reproducibility of the redundant target effect through the short version of Scarpina et al. (2018)’s task was done, because of the Italian lockdown. Thus, successive data collection, when the COVID-19 pandemic will be hopefully solved, should be necessary. I would underline that an online behavioral measurement cannot have the same level of accuracy than in any measurements performed in devoted and controlled experimental settings. Nevertheless, in the case of social restrictions as during the COVID-19 pandemic, online testing might represent a possible tool to verify larger samples’ psychological functioning. Finally, the sample collected in this study was heterogeneous, as traditionally observed in the case of online, and thus random, sampling. Nevertheless, it would be important to remark that no respondents reported COVID-19 symptoms at the time of the experiment or before. On the other hand, the majority of the respondents declared no symptoms, even though no clinical confirmation was available.

This preliminary study might offer a new perspective on the applicability of an online experiment focused on the facial emotion recognition ability to remotely assess the individuals’ psychological functioning, through a behavioral approach. The open-source nature of this task will easily allow its future application and updates. For example, although only fear was investigated here, all the other emotions, such as anger or sadness, can be assessed through the implicit facial emotion recognition task, as done in Scarpina et al. (2018). Thus, the *online* assessment and monitoring of the psychological well-being and emotional functioning, assessing both the object behavior (i.e., the way individuals act) through cognitive tasks *and* the subjective perception (i.e., the way individual think to act), through questionnaires, may be necessary, especially in the case of possible long-term maintenances of social restriction measurements. Notably, a higher exposition of others’ negative emotions may in turn impact on subjective emotional reactivity (the internal bodily signals, i.e., interoception, Craig, 2002), emotion recognition, and emotional regulation in terms of social cognition (Craig, 2002; Adolfi et al., 2017; Critchley and Garfinkel, 2017). As suggested by SARS and Ebola outbreaks (Maunder et al., 2003; Person et al., 2004; McMahon et al., 2016), fear toward the epidemic could have negative consequences in terms of adherence to social restrictions. This topic might be very relevant in the case of a gradual loosening of confinement measures, but with the maintenance of social restrictions and social distancing, as done in Italy from 2020 May 4th (i.e., “*Phase two*”), a situation that was described by the Italian Prime Minister Giuseppe Conte as an era “*of responsibility and coexistence with the virus*,” during a televised address to the Italians.

## Data Availability Statement

The datasets presented in this article are not readily available because: The restriction was related to the Ethical Approvement. Requests to access the datasets should be directed to FS, f.scarpina@auxologico.it.

## Ethics Statement

The studies involving human participants were reviewed and approved by Bioethics Commission of the University of Turin (istituito con D.R. n. 6502 del 23/10/2008), Università degli Studi di Torino. The patients/participants provided their informed consent to participate in this study.

## Author Contributions

FS performed the entire study and wrote the manuscript.

## Conflict of Interest

The author declares that the research was conducted in the absence of any commercial or financial relationships that could be construed as a potential conflict of interest.

## Footnotes

1 http://www.salute.gov.it/nuovocoronavirus

2 https://www.webosose.org/
